# Phage-encoded enzymes found in *Acinetobacter baumannii* convert pseudaminic acid to 8-epipseudaminic acid

**DOI:** 10.1038/s42003-025-08114-8

**Published:** 2025-05-05

**Authors:** Nowshin S. Sharar, Andrea Iovine, Cristina De Castro, Ruth M. Hall, Johanna J. Kenyon

**Affiliations:** 1 Centre for Immunology and Infection Control, School of Biomedical Sciences, Faculty of Health, Queensland University of Technology, Brisbane, QLD Australia; 2 Department of Chemical Sciences, University of Napoli Federico II Complesso Universitario Monte Santangelo, Via Cintia 4, I-80126 Naples, Italy; 3 School of Life and Environmental Science, The University of Sydney, Sydney, NSW Australia; 4 School of Pharmacy and Medical Sciences, Health Group, Griffith University, Gold Coast Campus, Southport, QLD Australia; 5 Institute for Biomedicine and Glycomics, Griffith University, Gold Coast Campus, Southport, QLD Australia

**Keywords:** Bacteriology, Bacterial evolution

## Abstract

The nonulosonic acid 8-epipseudaminic acid was discovered only recently in two *Acinetobacter baumannii* strains but the genes responsible for conversion of pseudaminic acid to its 8-epimer were not found at the K locus. Here, we use a pan-genome approach to identify a pair of carbohydrate biosynthesis genes, *epaA* and *epaB*, and demonstrate using NMR analysis of the capsular polysaccharide that they encode enzymes able to convert pseudaminic acid to its 8-epimer. Via an extensive survey of available *A. baumannii* genomes, we show that the *epaA* and *epaB* genes are present in 17 different *Caudoviricetes* class prophages. The prophages are in genomes that carry different capsule biosynthesis loci from isolates recovered in several different countries. The presence of *epaA* and *epaB* genes in *A. baumannii* isolates that are able to produce pseudaminic acid leads to modification of capsular polysaccharides that decorate their cell surface with potential implications for capsule typing and capsule-targeting therapies.

## Abstract (web summary)

The nonulosonic acid, 8-epipseudaminic acid, is produced from pseudaminic acid by EpaA and EpaB enzymes which are encoded by diverse prophages in genomes from geographically distributed *Acinetobacter baumannii* isolates.

## Introduction

**Fig. 1:**
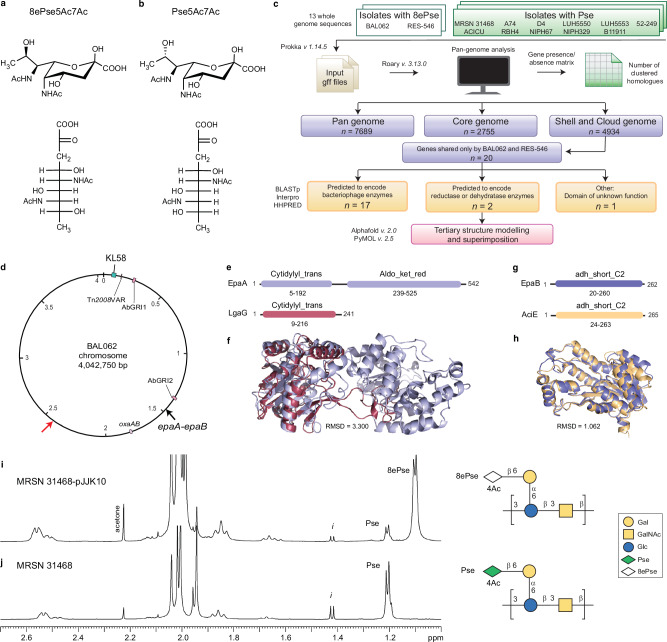
Identification and characterisation of *epaA-epaB.* Chemical structures of 8ePse5Ac7Ac (**a**) and Pse5Ac7Ac (**b**).Top: α-pyranose form; bottom: Fischer projection. **c** Comparative pan-genomics workflow used in this study. **d** Map of BAL062 chromosome (NCBI accession number LT594095.1) indicating location of KL58, *epaA-epaB* in Prophage 1 and resistance regions. Red arrow is sublineage C Prophage 3 location. Predicted protein domains in EpaA and LgaG (**e**), and EpaB and AciE (**g**). Modelled tertiary structures and superimposition of EpaA and LgaG (**f**) and EpaB and AciE (**h**). ^1^H-NMR profiles and CPS from MRSN 31468-pJJK10 (**i**) and wildtype (**j**) measured at 37 °C at 600 MHz. “*i*” is impurity.

Complex nine-carbon 5,7-diamino-3,5,7,9-tetradeoxy-non-2-ulosonic acids are a subfamily of non-2-ulosonic acids (NulOs) exclusively produced by bacteria, often pathogens, and therefore attract interest as potential targets for antimicrobial therapies^1^. Capsular polysaccharides (CPSs) produced by the critical priority pathogen *Acinetobacter baumannii*, are biological reservoirs for the largest number of NulOs, with legionaminic acid (Leg), 8-epilegionaminic acid (8eLeg), acinetaminic acid (Aci), 8-epiacinetaminic acid (8eAci) or pseudaminic acid (Pse) found as NulO constituents of some CPS types^2^. Moreover, 8-epipseudaminic acid (8ePse), representing the eighth isomer found in nature^3^, was recently discovered in the 5,7-di-acetylated form (8ePse5Ac7Ac; Fig. 1a) in the K135 CPS produced by isolate RES-546 from Russia^4^, and then in the K58 form from BAL062, a clonal complex 2 (CC2) reference isolate from Vietnam^5^. In both isolates, the K locus (KL) for CPS biosynthesis carries *psaA-F* genes for synthesis of the cytidine monophosphate (CMP)-activated form of Pse5Ac7Ac. However, MRSN 31468, an unrelated isolate that like BAL062 carries KL58, produces CPS containing Pse5Ac7Ac (Fig. 1b)^6^. Hence, we concluded that unknown genes outside the KL are likely required for 8-epimerization of Pse5Ac7Ac.

Here, we utilized a comparative pan-genomics approach to identify genes in BAL062 and RES-546, which were absent from the genomes of MRSN 31,468 and ten other isolates known to include Pse in their CPS. We identified two genes and demonstrated their role in converting Pse to 8ePse. We also examined the genetic context and global distribution of the identified genes in *A. baumannii* genomes.

## Results and discussion

### Identification of candidate gene(s) for the conversion of Pse to 8ePse

A comparative pan genomics approach (Fig. 1c) was used to identify gene candidates. Briefly, whole genome sequences for *A. baumannii* isolates BAL062 and RES-546, as well as all isolates (*n* = 11) with structural data reporting Pse in the CPS (see Table 1 for NCBI accession numbers and references), were used to construct a gene presence/absence matrix using the pan-genome analysis tool, *Roary v. 3.13.0*. These genomes were found to represent a total of 7689 genes, of which 2755 were present in all 13 genomes (core genome) and 4934 in 12 genomes or less (shell and cloud genome). Of 7689 total genes, 20 were shared by only BAL062 and RES-546 (Fig. 1c). The majority of these (*n* = 17) predict proteins associated with bacteriophages (Supplementary Table 1). One of the three remaining genes (#10 in Supplementary Table 1) was identified outside of detected prophage regions and encodes a product (GenPept accession number SBS23013.1) that includes a domain of unknown function (DUF6035). Given this sequence was found to be widespread in *Acinetobacter* genomes, and stronger candidates were identified (see below), it was not further investigated.

**Table 1:** NCBI accession numbers for whole genome sequences or reads used for Roary analysis

Isolate name	NCBI accession number	Genome reference	CPS type	Structure reference
BAL062^a^	GCA_900088705.1	^33^	K58	^5^
RES-546	GCA_023516475.1	^4^	K135	^4^
ACICU^a^	GCA_005519135.2	^34^	K2	^35^
A74	GCA_900118105.2	^36^	K2	^37^
RBH4	GCA_022869505.1	^38^	K6	^39^
D4	GCA_900476705.2	^40^	K16	^41^
NIPH67	GCA_000369265.1	^42^	K33	^43^
LUH5550	DRS005635	^44^	K42	^45^
NIPH329	GCA_000369225.1	^42^	K46	^46^
MRSN 31468	GCA_006491875.1	^23^	K58	^6^
LUH5553	DRS005657	^44^	K90	^47^
B11911	GCA_001077565.2	^48^	K93	^49^
52-249	GCA_044628175.1	^50^	K218	^50^

^a^Complete genome.

The two remaining genes (#17 and #18 in Supplementary Table 1) were found adjacent to each other and transcribed in the same direction. These genes were located ~1.4 Mb from the KL58 locus in the BAL062 chromosome (Fig. 1d), and as the gene products include reductase or dehydrogenase domains (see below) like other enzymes involved in the synthesis of *A. baumannii* non-2-ulosonic acids^7^, they were considered potential candidates involved in 8ePse synthesis and were respectively designated *epaA* and *epaB* for 8-epipseudaminic acid.

EpaA (542 amino acids (aa); GenPept accession number SBS22546.1) includes a CTP_transf_3 domain (IPR003329; formerly PF02348.22) at the N terminus (5-192 aa) and an Aldo_ket_red (IPR023210; PF00248.24) domain at the C terminus (239-525 aa) (Fig. 1e), suggesting a dual role as a cytidylyltransferase and a reductase. While EpaA shares no significant sequence identity with other enzymes known to be involved in NulO synthesis in *A. baumannii* (i.e. Psa, Leg, Aci or Ela enzymes), a structural relationship could be identified by superimposing tertiary structures of these enzymes modelled using Alphafold *v 2.3.2*. The N-terminus of EpaA aligned well (root mean square deviation (RMSD) score of 3.300) with LgaG (Fig. 1f; GenPept accession number AHB32583.2), the cytidyltransferase that activates Leg with CMP^7^.

EpaB (262 aa; GenPept accession number SBS22545.1) includes an adh_short_C2 (IPR002347; PF13561) domain (Fig. 1g) similarly found in oxidoreductases/dehydrogenases for NulO synthesis^7^. EpaB shares 41.5% aa identity with AciE (GenPept accession number ASR24079.1), an oxidoreductase (Fig. 1g) involved in the conversion of Aci to 8eAci^8,9^. Superimposition of the modelled tertiary structures (Fig. 1h) indicated a close structural relationship (RMSD 1.062). Taken together, these findings provided strong support that EpaA and EpaB direct conversion of Pse5Ac7Ac to 8ePse5Ac7Ac.

### Complementation of MRSN 31468

Attempts to delete the *epaA-epaB* genes from BAL062 using multiple recombineering approaches were unsuccessful. Hence, a 2576 bp sequence, including *epaA-epaB* with the intergenic region and short regions both immediately upstream and downstream, was amplified from BAL062 (NCBI accession number LT594095; bases 2549509-2552084) and inserted into a constructed *E. coli-A. baumannii* shuttle vector (see “Methods”). The resulting plasmid (designated pJJK10; Supplementary Fig. 1), and an equivalent plasmid lacking the *epaA-epaB* sequence (designated pJJK14, Supplementary Fig. 1), were independently introduced into MRSN 31468 via electroporation. The stability of these plasmids was validated via three subsequent rounds of subculture without selection. No significant differences in bacterial growth, colony morphology, or production of the CPS were identified between the MRSN 31468 wildtype and complemented forms carrying either pJJK14 or pJJK10 (Supplementary Fig. 1c-e; source data in Supplementary Fig. 5 and Supplementary Data 1). Hence, CPS from MRSN 31468 and MRSN 31468-pJJK10 were extracted for chemical analyses.

### CPS isolation and NMR analysis

CPS was isolated from dried cells of MRSN 31468-pJJK10 (2.7% (g/g) yield) and its proton spectrum was compared to that produced by the wildtype MRSN 31468. The ^1^H NMR spectra of the two CPSs shared many similarities (Supplementary Fig. 2; source data in Supplementary Data 2) and differed mostly in the high field region of the proton spectra. Indeed, the signal at 1.10 ppm, later assigned to **8eP**_**9**_, disclosed the presence of 8ePse in the CPS of MRSN 31468-pJJK10 (Fig. 1i; source data in Supplementary Data 2) together with a minor amount of Pse, inferred by the presence of the methyl, labelled as **P**_**9**_, at 1.20 ppm. The integration of these two signals returned a ratio of 1 to 6.4 between the two NulOs. Regarding the other signals, only some slight differences were observed in the carbinolic region (4.4–3.3 ppm) with the higher divergence occurring at ca. 4.4 ppm, while the anomeric region (5.1–4.4 ppm) had no substantial differences.

The identification of 8ePse in the CPS of MRSN 31468-pJJK10 was possible by analyzing the corresponding 2D NMR spectra (COSY, TOCSY and ^1^H-^13^C HSQC) according to established approaches^7^. Interestingly, the overlay of the HSQC spectrum (Supplementary Figs 3a–c; source data in Supplementary Data 2) with that recorded for the CPS of the wildtype returned a perfect match between the residues **A** – **C**. Since these have the same arrangement in the two CPSs, this enabled the identification of a Pse unit in MRSN 31468-pJJK10, that occurred as a minor substituent of the α-Gal unit **A**. Pse was partially acetylated at O-4 as found in the previous study^6^ and it was labelled as **P**^**Ac**^ (or **P**) depending on the presence (or absence) of this group. This preliminary assignment was counterchecked by analyzing the TOCSY and the COSY spectra (Table 2) and the successive analyses focused on the densities left unassigned. First, the region at 2.6–1.6 ppm (Supplementary Fig. 3c) reported the deoxy-protons linked to C-3 of the NulO; these densities slightly differed from those recorded for the Pse unit and the same pattern was observed for the other proton/carbon chemical shifts of the other positions of the pyranose ring, which indicated that the stereochemistry of the C-4 – C-6 atoms of this NulO was similar to that of Pse, except for the fact that this unit was also acetylated at O-4.

**Table 2:** ^1^H (600 MHz) and ^13^C (150 MHz) NMR attributions of the MRSN 31468-pJJK10 CPS measured at 37 °C

	1	2	3 (eq; ax)^§^	4	5	6;6’	7	8	9
A	4.99	3.85	3.97	3.86	4.05	4.04;3.62			
6-α-Gal	*99.2*	*69.5*	*70.5*	*70.6*	*70.6*	*65.2*			
B	4.78	4.07	3.92	4.13	3.71	3.82;3.75			
3-β-GalNAc	*102.9*	*52.8*	*81.0*	*69.2*	*75.9*	*62.3*			
C	4.55	3.41	3.68	3.64	3.66	3.99;3.74			
3,6-β-Glc	*105.5*	*74.1*	*85.1*	*69.2*	*74.9*	*66.8*			
P	–	–	2.50;1.66	3.94	4.21	3.92	4.14	4.18	1.20
β-Pse	*N.D*.	*N.D*.	*36.6*	*67.5*	*49.4*	*74.3*	*54.6*	*68.8*	*17.2*
P^Ac^	–	–	2.56;1.66	4.97	4.33	4.00	4.15	4.18	1.20
β-Pse4OAc	*N.D*.	*N.D*.	*33.6*	*70.1*	*46.6*	*73.7*	*54.6*	*68.8*	*17.2*
8eP	–	–	2.50;1.66	3.94	4.21	3.92	3.88	4.35	1.10
β-8ePse	*N.D*.	*N.D*.	*36.6*	*67.5*	*49.4*	*72.7*	*54.4*	*66.5*	*19.0*
8eP^Ac^	–	–	2.56;1.85	4.97	4.33	4.00	3.91	4.35	1.10
β-8ePse4OAc	*N.D*.	*N.D*.	*33.6*	*70.1*	*46.6*	*72.3*	*54.4*	*66.5*	*19.0*

*N.D.* “not detected”.

^§^ valid for the nonulosonic acids because these have two diastereotopic protons linked to C-3: “ax” indicates the axial position and “eq” the equatorial position.

All residues are in the pyranose form and the structure with each residue labelled is reported in Supplementary Fig. 2. The diagnostic areas of the HSQC and TOCSY spectra are displayed in Supplementary Fig. 3.

The inspection of the region at 1.3–1.0 ppm focused on the intense methyl signal 1.10 ppm, the H-9s of the NulO residue, which had a value different from that of Pse. This methyl group had one main correlation in the TOCSY spectrum with a proton at 4.35 ppm, attributed to H-8, and a second and weak one with a proton at 3.91 ppm, assigned to H-7 (Supplementary Fig. 3d), as confirmed by analyzing the COSY spectrum. No correlation from H-9 to H-6 was detected in contrast with what was found for the methyl group of the Pse unit (Supplementary Fig. 3d). Hence, the pattern noted in the TOCSY spectrum for the new NulO unit diverged from that of Pse, which suggested that it had the stereochemistry at C-8 inverted, namely that it was the epimer at C-8 of Pse. Accordingly, this unit was labelled **8eP** and this conclusion was supported by the fact that the ^3^J_H7,H8_ value reported for 8ePse is small (1.6 Hz) therefore not sufficient to carry out the magnetization propagation from H-9s up to H-6 in the TOCSY spectrum, as instead occurs for Pse (^3^*J*_H7,H8_ = 3.4 Hz)^10^.

In summary, the CPS isolated from *A. baumannii* MRSN 31468-pJJK10 presents two types of NulOs, with 8ePse being the most abundant and accounting ca. 85% of the total. Finally, 8ePse was partially acetylated at O-4 and by neglecting the contribution given by the Pse unit, the integration of the **8eP**^**Ac**^_**4**_ and **eP**_**4**_ densities in the HSQC spectrum determined that about 80% of the available positions were acetylated. Hence, NMR analyses revealed the presence of 8ePse5Ac7Ac in MRSN 31468-pJJK10 (Fig. 1i), replacing Pse5Ac7Ac in MRSN 31468 (Fig. 1j). 8ePse5Ac7Ac was 4-O-acetylated as was the Pse5Ac7Ac in the wildtype CPS as described previously^6^. This confirmed that EpaA-EpaB converts Pse5Ac7Ac to 8ePse5Ac7Ac.

### Genetic context of *epaA-epaB* in BAL062 and RES-546

**Fig. 2:**
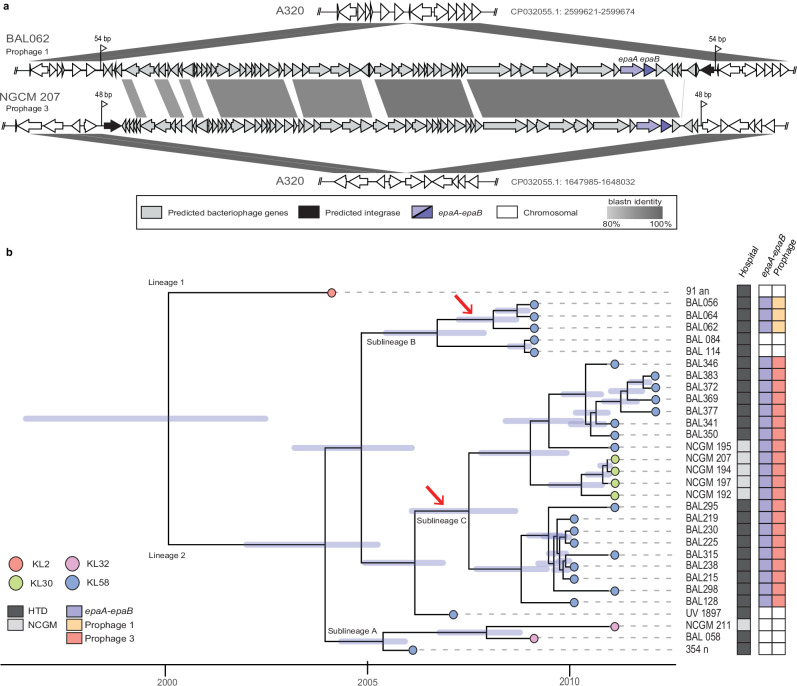
Prophage carrying *epaA-epaB* in *A. baumannii* CC2 isolates from Vietnam*.* **a** Comparison of A320 with Prophage 1 and 3 carrying *epaA-epaB* from BAL062 and NCGM207 (NCBI accession numbers in Supplementary Table 3). Grey shading is blastn identity with scale below. **b** Time-dated maximum likelihood core SNP phylogeny of CC2:L2 isolates from Vietnam. Node colours indicate KL. Dark grey boxes are HTD and light grey Cho Ray Hospital (NCGM). Blue boxes indicate *epaA-epaB* and prophage type is shown. Key is shown bottom left.

**Fig. 3:**
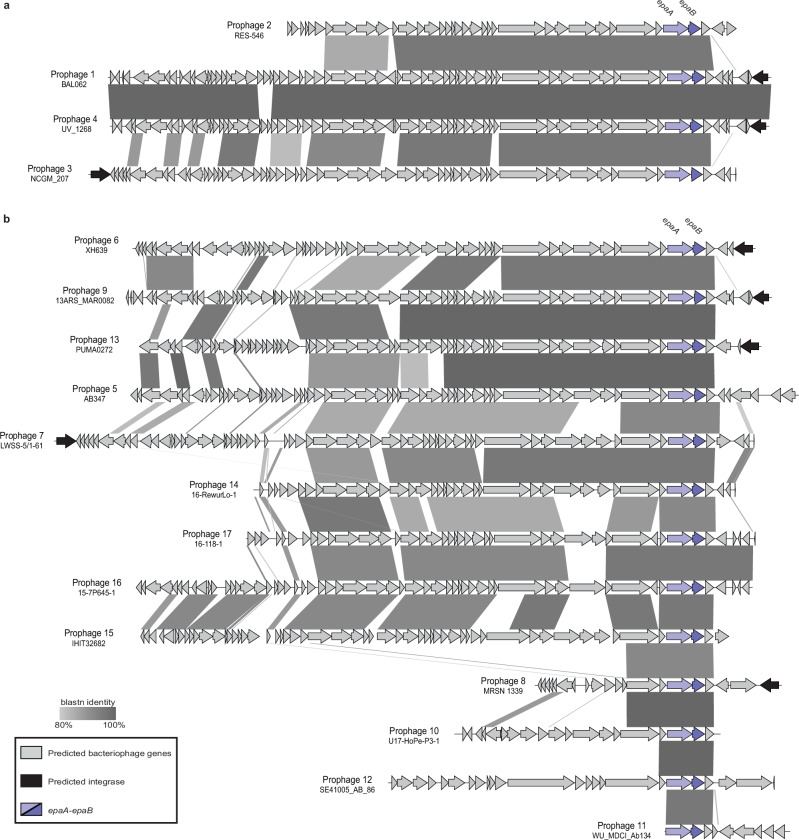
Comparison of prophage carrying *epaA-epaB* in *A. baumannii* genomes. **a** Comparison of Prophage 1–4. **b** Comparison of Prophage 5-17. Prophage number and name of representative strain (NCBI accession numbers in Supplementary Table 3) are shown on the left. ORFs are shown as arrows oriented by transcription direction and are coloured as per scheme shown below. Grey shading between sequences is blastn identity with scale below.

To examine the genetic context and determine how *epaA-epaB* had been acquired, the surrounding region from BAL062 was aligned with the A320 CC2 reference genome (NCBI accession number CP032055.1)^11^. This revealed a 43,065 bp insertion (LT594095.1, bases 2545250-2588314) in a tRNA-Gly gene (Fig. 2a). A tyrosine recombinase/integrase gene (locus tag BAL062_02452) was found at one of the termini, and a 54 bp duplication reconstituting the tRNA-Gly gene was detected. Further analysis revealed that *epaA-epaB* were part of an intact prophage (designated Prophage 1) in BAL062 that included a total of 62 predicted open reading frames (Supplementary Fig. 4) and belongs to the Class *Caudoviricetes*. In RES-546, *epaA-epaB* were in a 29.13 kb contig (WGS accession number JAMGSJ010000041.1) that shared 96% DNA identity (56% coverage) with part of the 43,065 bp BAL062 prophage 1 (Fig. 3a) in BAL062. However, the location of this prophage (Prophage 2) could not be identified using the available data.

### Distribution of *epaA-epaB* amongst *A. baumannii* isolates from Vietnam

BAL062 was recently reported to be a member of a phylogenetic sublineage (sublineage B of lineage 2)^5^ of an outbreak of CC2 isolates that occurred at the Hospital for Tropical Diseases (HTD) in Ho Chi Minh City, Vietnam during 2008–2012^12^. To examine phage-mediated acquisition in a regional population, short read data available for 145 CC2 isolates from Vietnam, either from the HTD outbreak^12^ or those reported in other studies^13,14^ were screened for *epaA-epaB* sequences using blastn against each NCBI Short Read Accession (SRA) number listed in those papers. Examination of a CC2 phylogeny of Vietnam isolates reported previously^13^ revealed that the *epaA-epaB* genes were restricted to some members of sublineage B and were present in all members of sublineage C recovered from HTD and Cho Ray Hospital (CRH), a second nearby hospital in Ho Chi Minh City, Vietnam. Isolates from CRH had been collected between 2011 and 2013^14^, which overlaps and follows the period of the HTD outbreak.

Reconstruction of the phylogeny with a select set of representative isolates from each sublineage (Fig. 2b) showed that the 43,065 bp Prophage 1 is in three of five total sublineage B isolates, including BAL062, indicating that it was a recent acquisition. For sublineage C members, *epaA-epaB* were in Prophage 3, a different 42,051 bp prophage (Fig. 2a) with *epaA-epaB* genes that share only 98.01% DNA identity with those from Prophage 1. In addition, Prophage 3 is inserted elsewhere in the chromosome (Fig. 2a and red arrow in Fig. 1d). Hence, the *epaA-epaB* genes had been acquired on at least two occasions. Additional analysis found that only one sublineage C isolate from CRH (labelled NCGM in Fig. 2b) carried KL58. For the nine other CRH isolates with Prophage 3, KL58 had been replaced by KL30, which does not include *psaA-F* genes to produce Pse, the predicted precursor for EpaA and EpaB.

We also screened the short read data for all other non-CC2 *A. baumannii* isolates recovered at the HTD reported previously^12^ and detected *epaA-epaB* in one further genome from a CC10:ST575 isolate, named UV_1268, that, like the CC2 isolates from this hospital, also carries KL58. The *epaA-epaB* genes in UV_1268 were found to be in a different 42,792 bp prophage (designated Prophage 4) that is closely related to Prophage 1 (Fig. 3a). These two prophage share 98% coverage with 99.99% nucleotide sequence identity, suggesting that they belong to the same species. The sequences differ in a short ~1 kb segment that includes open reading frames encoding proteins of unknown function. This CC10 isolate was recovered in 2005, which predates the 2008-2012 CC2 outbreak at HTD^12^ that includes BAL062^5^. Hence, KL58 and Prophage 4 may have been acquired by a CC2 isolate prior to the outbreak, with a small recombination event leading to the generation of Prophage 1.

Prophage 3 from sublineage C was detected in an additional two genomes from clinical isolates also recovered from CRH during the same period as the CC2 isolates (Supplementary Table 2). Both isolates belong to ST215 (alleles *cpn60*-27, *fusA*-2*, gltA*-7*, pyrG*-2*, recA*-2*, rplB*-1*, rhoB*-2), which is a triple locus variant of ST2, and hence may be related. These isolates both carry KL60, which again does not include the *psaA-F* genes and therefore cannot produce 8ePse because the Pse precursor is missing.

### Global distribution of prophages 1, 3 and 4

A total of 27,079 publicly available *A. baumannii* whole genome sequences were searched for the presence (100% coverage with >98% nucleotide sequence identity) of Prophages 1, 3 and 4 found in isolates originating in Vietnam. Prophage 1 from BAL062 was not detected in any further genome sequences, indicating geographical restriction to Vietnam. However, Prophage 4, found in the CC10:ST575:KL58 isolate from Vietnam (UV_1268, described above), was also found in one ST309:KL169 environmental isolate from China and one ST1554 environmental isolate from Germany that carries a KL with no detectable match in the *Kaptive* database (Supplementary Table 2). The KL in both these isolates include *psa* genes for synthesis of Pse.

Prophage 3 from sublineage C was detected in an additional 10 genomes from clinical isolates recovered in Egypt in either 2020 or 2021 (Supplementary Table 2). These belong to ST158 and carry either KL6 (*n* = 3), KL23 (*n* = 5) or KL49 (*n* = 2), though only KL6 and KL23 sequences include *psaA-F* genes.

### Global distribution of *epaA-epaB*

**Fig. 4:**
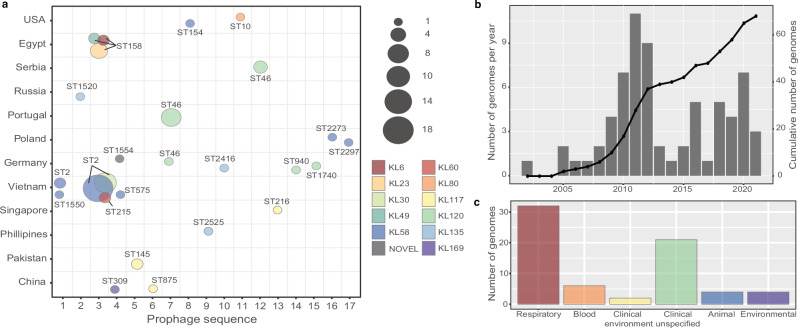
Distribution of prophage carrying *epaA-epaB* in *A. baumannii.* **a** Bubble scatterplot constructed using ggplot2 showing distribution of *epaA-epaB* by prophage (*x*-axis) and geographical location (*y*-axis). Sequence types (STs) are indicated. Colours represent KL type and bubble size is number of genomes. Scheme shown to right. **b** Number of genomes (*left* axis) and cumulative number of genomes (*right* axis) per collection year. **c** Number of genomes per isolation source. Source data for all charts is provided in Supplementary Table 2.

We further screened the 27,079 publicly available genomes for the presence of *epaA-epaB* in other genetic contexts and detected *epaA-epaB* in a further 23 genomes (Supplementary Table 2). Together with BAL062, RES-546 and the Vietnam isolates, the total number included 68 isolates representing 21 sequence types (STs) from 12 countries (Fig. 4a). These isolates had been recovered between 2002 and 2021 (Fig. 4b) and were mostly from clinical respiratory sources (Fig. 4c). A total of 12 K loci were identified, only nine of which include *psaA-F* genes. In all cases, *epaA-epaB* were found in prophage regions, and a total of 13 further prophage were identified (numbered 5-17; Fig. 3b), all of which are predicted to be of the class *Caudoviricetes* (Supplementary Table 3). The *epaA-epaB* genes in Prophages 1 and 4 share 100% DNA identity (100% coverage) with those in Prophages 9 and 10. For the remaining prophage, *epaA-epaB* genes shared 97.97-99.92% identity with *epaA-epaB* in Prophage 1. Most prophage appeared sporadic. Hence, only Prophage 3 and 4 were found in isolates with different KL, belonging to different STs recovered from different countries (Fig. 4a).

The conversion of Pse5Ac7Ac to 8ePse5Ac7Ac directed by enzymes encoded by *epaA-epaB* genes found in *A. baumannii* prophage genomes may affect the efficacy of new antibody or phage therapies that target the CPS. However, because the phages are circulating independently and because replacement of the K locus is common, especially in the CC2 clonal complex that includes the majority of extensively antibiotic resistant *A. baumannii* isolates, the *psaA-F* genes to produce the Pse5Ac7Ac precursor are not always present in isolates that carry the *epaA-epaB* genes.

### Detection of *epaA-epaB* genes using *Kaptive*

As phage-mediated acquisition of *epaA-epaB* genes in isolates carrying KL with *psaA-F* can lead to structural diversity in the CPS, *epaA-epaB* detection is needed in order to select the most appropriate CPS-targeting therapy for treatment of infections that are resistant to clinically available antibiotics. To facilitate in silico detection of *epaA-epaB* in *A. baumannii* genomes, the *Kaptive v 2.0.7* database for identification of CPS biosynthesis genes^15,16^ was updated to include the *epaA-epaB* sequence (see “Methods”). *Kaptive v 2.0.7*, as well as the updated database and special logic files for typing *A. baumannii* KL and detecting *epaA-epaB*, is available at https://github.com/klebgenomics/Kaptive/tree/master/reference_database.

## Methods

### Identification of genes required for 8-epimerisation of Pse5Ac7Ac

Whole genome sequences for the 13 *A. baumannii* isolates used for comparative pan-genomics analysis were either downloaded directly from NCBI or assembled from short read data, with NCBI accession numbers are listed in Table 1. Input gff files were obtained using Prokka *v 1.14.5*^17^ and submitted to Roary *v. 3.13.0*^18^ to produce a gene presence/absence matrix using a homology cut off value of 85% aa identity. Coding sequences shared by only BAL062 and RES-546 (*n* = 20) were assessed using BLASTp (https://blast.ncbi.nlm.nih.gov/Blast.cgi), Interpro (https://www.ebi.ac.uk/interpro/) and HHPRED. Sequences for EpaA and EpaB were submitted to AlphaFold2.0^19^ via the Galaxy Australia platform^20^ to produce tertiary structure models using default parameters. PyMOL2.5 (https://www.pymol.org/) was used to visualize and superimpose modelled tertiary structures, with RMSD scores calculated using the *align* command. Prophage class classification and open reading frame detection was performed using PhageScope^21^. Chromosome map was constructed using SnapGene *v* 7.2.1 (snapgene.com).

### Bacterial strains and cultivation

We thank Associate Professor Amy Cain for providing *A. baumannii* isolate BAL062, which is a CC2:ST1550:KL58 carbapenem resistant isolate recovered at the HTD in Ho Chi Minh City, Vietnam in 2009^22^, and is used as a reference isolate^5^. We thank Dr Patrick McGann for providing *A. baumannii* isolate MRSN31468, which is an ST154:KL58 isolate that is commercially available from the diverse panel of *A. baumannii* isolates^23^. Bacterial isolates were routinely cultured in Luria-Bertani medium at 37 °C with or without shaking at 200 rpm. Plasmids were maintained using 100 μg/ml ampicillin or 50 μg/ml apramycin (Sigma Aldrich) where appropriate for selection in *A. baumannii* MRSN 31468. Optical density (OD) was measured by absorbance (600 nm) using a densitometer.

### Vector construction and complementation of MRSN 31468

Polymerase chain reaction (PCR) using Phusion HotStart II DNA Polymerase (ThermoFisher Scientific) was used to amplify the *aacC4* apramycin resistance gene from pSRC119-A/C (GenBank accession number KM670336.1), the *Acinetobacter-*specific *ori* from pWH1266^24^ and the 2576 bp *epaA-epaB* sequence from BAL062 whole cell genomic DNA. Primers (listed in Supplementary Table 4) included sequence overlapping with pUC19 or adjacent amplicons in the final constructs. PCR conditions were as follows: 98 °C for 30 s, 98 °C for 10 s, 50 °C for 30 s, 72 °C for 3 min (34 cycles), and a final extension at 72 °C for 5 min. Products were purified using PureLink™ PCR purification kit (Invitrogen, ThermoFisher Scientific) and DNA concentration was measured using Qubit fluorometric quantification assay.

First, purified pUC19 was digested with *Ssp*I (ThermoFisher Scientific) and ligated with *aacC4* using the GeneArt Gibson assembly cloning kit (ThermoFisher Scientific) to insert this gene immediately upstream of *bla* under control of the same promoter. This construct was then double digested with *Bam*HI and *Kpn*I, purified then amplified with specific reverse oriented primers (Supplementary Table 4) located at the ends of *lacZ’*. The product was treated with *Dpn*I to reduce circular template carryover, then joined with purified amplicons (*ori*, or *ori* and *epaA-epaB*) using the GeneArt Gibson assembly cloning kit (ThermoFisher Scientific) to create vectors pJJK10 and pJJK14 (Supplementary Fig. 1). Vectors were confirmed by whole plasmid sequencing at Plasmidsaurus (US). Plasmid DNA (100 ng) was added to electrocompetent cells of MRSN 31468 subcultured 1:100 and harvested at late-logarithmic phase (OD_600nm_ ~ 0.7), then pulsed at 1.8 kV with a Bio-Rad GenePulser. Cells were recovered at 37 °C for ~2 h, and then selected with 50 μg/ml apramycin incubated at 37 °C overnight.

### Colony morphology and bacterial growth analyses

Growth of MRSN 31468-pJJK10 and MRSN 31468-pJJK14 in liquid culture was compared with the wildtype by measuring bacterial growth every 30 min for 24 h at OD600 in a 96-well plate using a CLARIOstar plate reader. Colony morphology was compared by inoculating 5 μl of overnight culture onto Luria-Bertani agar, incubating for 6 days at 37 °C, and photographing under white light using a ChemiDoc XRS gel imaging system.

### Statistics and reproducibility

For bacterial growth curves, measurements for *n* = 3 biologically independent samples were used. Error bars indicate the standard deviation from the mean as shown in Supplementary Fig. 1e.

### Extraction and visualisation of CPS

Visualisation of ‘attached’ CPS closely associated with the cell surface and ‘shed’ CPS that has shed into the extracellular milieu was performed using SDS-PAGE with Alcian blue staining^25^. Briefly, 10 mL overnight cultures were centrifuged at 4000 rpm at 4 °C for 15 min to pellet cells. Shed CPS was extracted from supernatant fractions by precipitation in 4 × volumes of ice-cold 100% ethanol at −20 °C overnight. Shed CPS samples were then resuspended in sterile milli-Q water following centrifugation at 12,000 rpm at 4 °C for 15 min.

Attached CPS was purified from the cell fractions (pellets) of the same overnight cultures by first resuspending pellets in 200 µL TAE, then adding 400 µL Lysis Buffer (100 mM SDS, 50 mM Tris, 0.128 mM NaCl) followed by 600 µL phenol:chloroform:isoamylalcohol (25:24:1). Samples were vortexed vigorously then incubated at 65 °C for 15 min. The aqueous phase was separated by centrifugation at 5 °C for 15 min, transferred to fresh tubes, then 200 µL Milli-Q water, 50 µL 3 M sodium acetate (pH 5.2), and 1 mL ice-cold 100% ethanol added. Samples were precipitated at –80 °C for 15 min, centrifuged for 5 °C for 15 min, then the pellets resuspended in 50 µL Milli-Q water. Samples were then incubated with 3 µL DNase (10 mg/mL) and 3 µL RNase (10 mg/mL) at 37 °C for 45 min, followed by incubation at 56 °C for 1 h with 5 µL Proteinase K (20 mg/mL). An equal volume of phenol:chloroform:isoamylalcohol (25:24:1) was added, and the samples vortexed vigorously. Samples were centrifuged again for 15 min at 5 °C, then 193 µL 50 mM Tris, 7 µl 3 M sodium acetate (pH 5.2) and three volumes of 100% ice-cold ethanol added to the pellets then incubated at –80 °C for 15 min. Samples were then centrifuged at 5 °C for 30 min, and pellets resuspended in 50 µL Milli-Q water.

Equivalent volumes of purified shed and attached CPS were mixed with loading buffer then loaded onto SDS-PAGE tricine gels (4% stacking and 16% separating). SDS-PAGE was performed with cathode buffer (1.0 M Tris-HCl, 1.0 M Tricine, 1% SDS pH 8.25) in the inner tank and anode buffer (2.0 M Tris-HCl, pH 8.9) in the outer tank, and electrophoresed at 50 V for 30 min and then at 150 V for 1 hr. Gels were fixed for 1 h in fixative solution containing 25% isopropanol and 7% acetic acid, then stained overnight in fixative solution with 0.05% w/v Alcian blue added. Gels were decolourised for 30 min in fixative solution, then imaged using a ChemiDoc XRS gel imaging system.

### CPS isolation

Cells of MRSN 31468-pJJK10 were treated according to the PCP method^26^ to remove the lipooligosaccharide and later extracted using the hot water/phenol method^27^ to yield a crude capsular polysaccharide (442 mg, yield 8.2% g/g_cells_). This crude product (45 mg) was further purified by enzymatical treatment with DNAse, RNAse and finally proteinase K, then it was dialyzed (cut-off 12–14,000 Da) and freeze-dried, to yield to the pure CPS later used for NMR studies (10 mg, 2.7% g/g_cells_)^28^. CPS from MRSN 31468 wildtype was isolated by the same procedure described in the previous study^6^.

### Sample preparation and NMR acquisition conditions

MRSN 31468-pJJK10 CPS (7 mg) was treated with 0.5 ml of Dowex 50 W X8 resin suspended in water to convert the carboxylic function of the nonulosonic residue in the H^+^ form. After filtering the solution from the resin beads and freeze-drying, the CPS was solved in 550 ml of 99.5% D_2_O to record the NMR spectra. COSY, TOCSY and HSQC NMR spectra were measured on a Bruker Avance II 600 MHz spectrometer equipped with a cryo-probe set 37 °C, using acetone (δ_H_ 2.225, δ_C_ 31.45) as internal reference for calibration. A 100 ms mixing time was set for the spin-lock during the TOCSY acquisition. Two-dimensional NMR spectra were measured by using Bruker TopSpin 3.5 programme. The spectra were processed and analyzed with the same software.

### Maximum-likelihood core-SNP phylogeny

A core-SNP phylogeny was created using available short read data (SRA accession numbers listed in Supplementary Table 5) for CC2 isolates recovered from Vietnam. Reads were downloaded from the European Nucleotide Archive (ENA) and mapped onto the complete genome sequence for BAL062 (NCBI accession number LT594095.1) using the nf-core/bactmap *v 1.0.0* pipeline (https://nf-co.re/bactmap/1.0.0/usage). The pipeline removed regions of recombination using Gubbins, called variant SNPs using SNP sites, then constructed a maximum-likelihood phylogeny using RAxML-NG with default parameters. Bayesian analysis was performed with the tool BactDating^29^ using default parameters. The Gubbins recombination-corrected tree and years of collection were used as inputs, and 10^5^ iterations were used for Markov chain Monte Carlo (MCMC) settings. The resulting tree was annotated using Adobe Illustrator.

### Distribution analysis

A total of 27,079 whole genome sequences available in NCBI under the *A. baumannii* ID:470 taxonomical classification (as of 21st May 2024) were screened for the presence of *epaA-epaB* using BLASTn (https://blast.ncbi.nlm.nih.gov/Blast.cgi). Metadata (strain name, host, geographical location/country, collection date) available under the Biosample accession numbers for each genome assembly positive for *epaA-epaB* genes was obtained using metatools_ncbi (www.github.com/farhat-lab/metatools_ncbi). Command-line *Kaptive v 2.0.7* was used to determine the sequence at the K locus using the most recent iteration of the *A. baumannii* KL reference database that includes 241 KL^15^. Multilocus sequence typing (MLST) was performed using the *mlst* tool (https://github.com/tseemann/mlst) with the Institut Pasteur scheme available at https://pubmlst.org/organisms/acinetobacter-baumannii. Distribution analyses were visualised with ggplot2^30^ and/or tidyverse^31^ packages in RStudio *v. 3.3.0* + , and then annotated in Adobe Illustrator. The geom_point function with the position_jitter option was specifically used through ggplot2 to construct the bubble scatterplot. Prophage regions were visualised and compared using EasyFig *v* 2.2.5^32^.

### Update to *Kaptive*

The *Kaptive* reference sequence database for in silico *A. baumannii* CPS prediction^15,16^ was updated to include the sequence for *epaA-epaB* from BAL062. The special logic file that operates in conjunction with this database was also updated to enable prediction K58-EpaAB or K135-EpaAB upon detection of KL58 or KL135 with *epaA-epaB* in the same genome. The updated *Kaptive* database and special logic file for *A. baumannii* CPS were uploaded to https://github.com/klebgenomics/Kaptive/tree/master/reference_database.

### Reporting summary

Further information on research design is available in the Nature Portfolio Reporting Summary linked to this article.

## Supplementary information

Supplementary Information

Supplementary Data 1

Supplementary Data 2

Description of Additional Supplementary Files

Reporting Summary

## Data Availability

The *Kaptive* reference sequence database and special logic file for in silico *A. baumannii* CPS prediction updated as part of this study is available at https://github.com/klebgenomics/Kaptive/tree/master/reference_database. Source data for Fig. S1 gels (uncropped/unedited gels) is provided in Supplementary Fig. 5. Source data for Fig. S1 growth curves is provided in Supplementary Data 1. Source data including raw NMR data is provided in Supplementary Data 2. Annotated plasmid sequences were deposited in Benchling and are available via the follow links: https://benchling.com/s/seq-VS39HOaeHjdQrkJJMVWc?m=slm-487h4o6DdEROyNjhA1cV (pJJK14) and https://benchling.com/s/seq-Gwada9ELt4yWqcWmCJce?m=slm-Rd1B0D4RplCYNG1IzS5Y (pJJK10).

## Code availability

The study did not involve the creation of code for bioinformatics analyses. All the used software is freely accessible and is referenced in the methods.
