# Supplementary material for: Phage-encoded enzymes found in Acinetobacter baumannii convert pseudaminic acid to 8-epipseudaminic acid
Source: Commun Biol. 2025 May 5;8:700. doi: 10.1038/s42003-025-08114-8 (PMC12053666; doi:10.1038/s42003-025-08114-8)
Supplement: Supplementary file 1 — Supplementary Information [file 42003_2025_8114_MOESM1_ESM.pdf]

Supplementary Information

Phage-encoded enzymes found in *Acinetobacter baumannii* convert pseudaminic acid to 8-epipseudaminic acid

Nowshin S. Sharar, Andrea Iovine, Cristina De Castro, Ruth M. Hall, Johanna J. Kenyon

Table of Contents

SUPPLEMENTARY TABLES

Supplementary Table 1. Analysis of the 20 coding sequences shared by BAL062 (GCA_900088705.1) and RES-546 (GCA_023516475.1) genomes

Supplementary Table 2. Information and assembly accession numbers for isolates carrying *epaA*-*epaB*.

Supplementary Table 3. Details of the prophage carrying *epaA*-*epaB* detected in *A. baumannii* genomes.

Supplementary Table 4. Primers used in this study.

Supplementary Table 5. SRA numbers used for phylogenetic analyses

SUPPLEMENTARY FIGURES

Supplementary Figure 1. Cloning vectors and effect of bacterial growth and CPS production.

Supplementary Figure 2. ^{1}H-NMR profiles and structures of the capsular polysaccharides from *A. baumannii* strains BAL062 and MRSN 31468 measured at 37°C at 600 MHz.

Supplementary Figure 3. 2D NMR spectra recorded for the CPS produced by *A. baumannii* MRSN 31468-pJJK10 and comparison (by overlay) with the spectra of the CPS from MRSN31468 (wildtype).

Supplementary Figure 4. Annotation of Prophage 1 genome detected in the BAL062 chromosome.

SUPPLEMENTARY TABLES

Supplementary Table 1. Analysis of the 20 coding sequences shared by BAL062 (GCA_900088705.1) and RES-546 (GCA_023516475.1) genomes

Coding sequence	Base position in LT594095.1	Locus tag	Protein_id	Size (aa)	Annotation in BAL062 (GCA_900088705.1)	Protein domain(s) and/or family	Significant BLASTp hit outside *Acinetobacter* (%aa identity)	PHROGS
1	962272-962781	BAL062_03034	SBS23112.1	169	Uncharacterised protein	-	hypothetical protein Ab1052phi_05 [Acinetobacter phage Ab105-2phi]; ALJ99100.1; 85.53%	phrog_31407; unknown function
2	966566-966826	BAL062_03026	SBS23104.1	86	Uncharacterised protein	-	hypothetical protein Acba3_027 [Acinetobacter phage Acba_3]; WCF71507.1; 62.34%	phrog_19948; unknown function; phrog_14634; DNA binding protein; DNA, RNA and nucleotide metabolism
3	966912-967715	BAL062_03025	SBS23103.1	267	Uncharacterised conserved protein,Uncharacterised conserved protein (DUF2303)	PF10065.12 (DUF2303)	DUF2303 family protein [Klebsiella pneumoniae]; MDW7450494.1; 99.00%	phrog_210; unknown function; phrog_11501; Immunoglobulin/Fibronectin type III/E set domains/PapD-like; CueP; Putative periplasmic or exported protein
4	967756-968088	BAL062_03024	SBS23102.1	110	hypothetical protein	-	hypothetical protein [Klebsiella pneumoniae]; MBL2055253.1; 100%	phrog_204; unknown function; phrog_997; replication terminator; DNA, RNA and nucleotide metabolism
5	969433-969666	BAL062_03021	SBS23099.1	77	Helix-turn-helix	PF01381.25 (HTH_3); PF04545.19 (Sigma70_r4); PF13412.9 (HTH_24)	XRE family transcriptional regulator [Klebsiella pneumoniae]; MBL2055238.1; 100%	phrog_23834; tetra-helical, lambda repressor-like
6	980874-983162	BAL062_03001	SBS23079.1	246	Uncharacterised protein	-	hypothetical protein [Caudoviricetes sp.]; DAL29797.1; 100%	phrog_19541; MtlR-like; MtlR; Mannitol operon repressor
7	983170-983991	BAL062_03003	SBS23081.1	273	hypothetical protein	-	hypothetical protein [Caudoviricetes sp.]; DAL29795.1; 100%	phrog_25984; unknown function; phrog_21283; LuxS/MPP-like metallohydrolase; Peptidase_M16_C_1
8	985338-986282	BAL062_03000	SBS23078.1	314	Abi family protein,Abi-like protein	PF07751.14 (Abi_2)	putative bacteriophage abortive infection protein [Vibrio tapetis]; ACB99648.1; 52.22%	phrog_26181; unknown function; phrog_2158; abortive infection resistance protein; moron, auxiliary metabolic gene and host takeover
9	1001541-1002167	BAL062_02981	SBS23059.1	208	Uncharacterised protein	-	hypothetical protein vBAbaMABMM1_09 [Acinetobacter phage vB_AbaM_ABMM1]; WEU68029.1; 75.48%	phrog_5216; Periplasmic domain of ExbD/TolR; ExbD_3

10	1054131-1055513	BAL062_02934	SBS23013.1	460	hypothetical protein	PF19500.2 (DUF6035)	Uncharacterised protein [Klebsiella pneumoniae]; SSW83143.1; 60.25%	-
11	1263963-1264352	BAL062_02719	SBS22799.1	129	Uncharacterised protein	-	hypothetical protein [Acinetobacter phage A785.1]; WMT10770.1; 99.22%	phrog_7138; unknown function
12	1483085-1483726	BAL062_02467	SBS22553.1	213	Uncharacterised protein	PF09343.13 (DUF2460)	Uncharacterised conserved protein [Acinetobacter phage MD-2021a]; CAH1069037.1; 56.22%	phrog_2913; Phage tail protein, glycoside hydrolase
13	1483723-1484580	BAL062_02466	SBS22552.1	285	hypothetical protein	PF09931.12 (DUF2163); PF09356.13 (Phage_BR0599)	Uncharacterised conserved protein [Acinetobacter phage MD-2021a]; CAH1069033.1; 77.19%	phrog_778; Phage tail protein
14	1484639-1486393	BAL062_02465	SBS22551.1	584	hypothetical protein	PF13088.9 (BNR_2)	Neuraminidase (sialidase) [Acinetobacter phage MD-2021a]; CAH1069029.1; 89.38%	phrog_35653; tail fiber protein; putative tailspike protein
15	1486371-1487282	BAL062_02464	SBS22550.1	303	Uncharacterised protein	-	Uncharacterised protein [Acinetobacter phage MD-2021a]; CAH1069025.1; 88.45%	phrog_28256; Concanavalin A-like lectins/glucanases; Laminin_G_3
16	1487722-1490211	BAL062_02462	SBS22548.1	829	fibronectin type III domain protein,Fibronectin type III domain	PF13550.9 (Phage-tail_3)	host specificity protein [Acinetobacter phage MD-2021a]; CAH1069017.1; 77.13%	phrog_4271; C-terminal insertion domain in phage tail proteins
17	1490759-1492387	BAL062_02460	SBS22546.1	542	3-deoxy-manno-octulosonate cytidylyltransferase	PF00248.24 (Aldo_ket_red); PF02348.22 (CTP_transf_3)	Spore coat polysaccharide biosynthesis protein SpsF, cytidylyltransferase family [Kosakonia radicincitans]; SKC22710.1; (54.07%)	phrog_8735; Aldo_ket_red; phrog_3980; Nucleotide-diphospho-sugar transferases; NTP_transfer
18	1492390-1493178	BAL062_02459	SBS22545.1	262	short chain dehydrogenase,Rhamnolipids biosynthesis 3-oxoacyl-[acyl-carrier-protein] reductase,gluconate 5-dehydrogenase,Uncharacterised conserved protein,2-deoxy-D-gluconate 3-dehydrogenase,short chain dehydrogenase	PF13561.9 (adh_short_C2); PF00106.28 (adh_short)	SDR family oxidoreductase [Mangrovibacter plantisponsor]; WP_110024859.1; (59.16%)	phrog_3142; FabG-like 3-oxoacyl-(acyl-carrier-protein) reductase; moron, auxiliary metabolic gene and host takeover;
19	2799772-2801010	BAL062_01185	SBS21293.1	412	Putative Phage integrase,Putative prophage CPS-53 integrase,integrase,Site-specific recombinase XerC,Phage integrase family	PF00589.25 (Phage_integrase)	tyrosine-type recombinase/integrase [Acinetobacter phage YMC11/11/R3177]; YP_009593338.1; (97.73%)	phrog_1; integrase; integration and excision
20	2841805-2842074	BAL062_01122	SBS21230.1	89	Uncharacterised protein	-	putative excisionase [Acinetobacter phage AM106]; AWD93177.1; (87.50%)	phrog_34998; Putative DNA-binding domain

Supplementary Table 2. Information and assembly accession numbers for isolates carrying *epaA-epaB*.

Isolate name	Accession No.	Country: City/Region	Year	Source category	Isolation source	CC	ST	KL	Prophage
BAL062	GCA_900088705.1	Vietnam: Ho Chi Minh	2009	Respiratory	VAP	2	1550	KL58	Ph1
BAL056	ERR190414	Vietnam: Ho Chi Minh	2009	Respiratory	VAP	2	2	KL58	Ph1
BAL064	ERR190417	Vietnam: Ho Chi Minh	2009	Respiratory	VAP	2	2	KL58	Ph1
RES-546	GCA_023516475.1	Russia: Irkutsk	2002	Clinical unspecified	intra-abdominal	-	1520	KL135	Ph2
BAL128	ERR190425	Vietnam: Ho Chi Minh	2010	Respiratory	VAP	2	2	KL58	Ph3
BAL215	ERR190448	Vietnam: Ho Chi Minh	2010	Respiratory	VAP	2	2	KL58	Ph3
BAL219	ERR190450	Vietnam: Ho Chi Minh	2010	Respiratory	VAP	2	2	KL58	Ph3
BAL225	ERR190452	Vietnam: Ho Chi Minh	2010	Respiratory	VAP	2	2	KL58	Ph3
BAL230	ERR190454	Vietnam: Ho Chi Minh	2010	Respiratory	VAP	2	2	KL58	Ph3
BAL238	ERR190457	Vietnam: Ho Chi Minh	2010	Respiratory	VAP	2	2	KL58	Ph3
BAL295	ERR190473	Vietnam: Ho Chi Minh	2011	Respiratory	VAP	2	2	KL58	Ph3
BAL298	ERR190474	Vietnam: Ho Chi Minh	2011	Respiratory	VAP	2	2	KL58	Ph3
BAL315	ERR190478	Vietnam: Ho Chi Minh	2011	Respiratory	VAP	2	2	KL58	Ph3
BAL341	ERR190490	Vietnam: Ho Chi Minh	2011	Respiratory	VAP	2	2	KL58	Ph3
BAL346	ERR190491	Vietnam: Ho Chi Minh	2011	Respiratory	VAP	2	2	KL58	Ph3
BAL350	ERR190493	Vietnam: Ho Chi Minh	2011	Respiratory	VAP	2	2	KL58	Ph3
BAL369	ERR190498	Vietnam: Ho Chi Minh	2012	Respiratory	VAP	2	2	KL58	Ph3
BAL372	ERR190499	Vietnam: Ho Chi Minh	2012	Respiratory	VAP	2	2	KL58	Ph3
BAL377	ERR190500	Vietnam: Ho Chi Minh	2012	Respiratory	VAP	2	2	KL58	Ph3
BAL383	ERR190501	Vietnam: Ho Chi Minh	2012	Respiratory	VAP	2	2	KL58	Ph3
NCGM 350	GCA_016498805.1	Vietnam: Ho Chi Minh	2013	Clinical unspecified		2	2	KL30	Ph3
NCGM 351	GCA_016498825.1	Vietnam: Ho Chi Minh	2013	Clinical unspecified		2	2	KL30	Ph3
NCGM 330	GCA_016499725.1	Vietnam: Ho Chi Minh	2012	Clinical unspecified		2	2	KL30	Ph3
NCGM 329	GCA_016499785.1	Vietnam: Ho Chi Minh	2012	Clinical unspecified		2	2	KL30	Ph3
NCGM 307	GCA_016501185.1	Vietnam: Ho Chi Minh	2012	Clinical unspecified		2	2	KL30	Ph3
NCGM 207	GCA_016501725.1	Vietnam: Ho Chi Minh	2011	Clinical unspecified		2	2	KL30	Ph3
NCGM 197	GCA_016502925.1	Vietnam: Ho Chi Minh	2011	Clinical unspecified		2	2	KL30	Ph3
NCGM 194	GCA_016502945.1	Vietnam: Ho Chi Minh	2011	Clinical unspecified		2	2	KL30	Ph3
NCGM 195	GCA_016503035.1	Vietnam: Ho Chi Minh	2011	Clinical unspecified		2	2	KL58	Ph3
NCGM 192	GCA_016503165.1	Vietnam: Ho Chi Minh	2011	Clinical unspecified		2	2	KL30	Ph3
LBMM 6411	GCA_030516295.1	Egypt: Alexandria	2021	Blood	blood	-	158	KL23	Ph3
LBMM 6413	GCA_030516315.1	Egypt: Alexandria	2021	Blood	blood	-	158	KL23	Ph3
LBMM 6408	GCA_030516385.1	Egypt: Alexandria	2020	Respiratory	BAL	-	158	KL6	Ph3
LBMM 6410	GCA_030516395.1	Egypt: Alexandria	2021	Clinical unspecified	swab	-	158	KL6	Ph3
LBMM 6409	GCA_030516415.1	Egypt: Alexandria	2020	Respiratory	BAL	-	158	KL6	Ph3

LBMM 6405	GCA_030516465.1	Egypt: Alexandria	2020	Respiratory	BAL	-	158	KL23	Ph3
LBMM 6403	GCA_030516485.1	Egypt: Alexandria	2020	Respiratory	BAL	-	158	KL23	Ph3
LBMM 6400	GCA_030516525.1	Egypt: Alexandria	2020	Clinical unspecified	Tissue	-	158	KL23	Ph3
LBMM 6389	GCA_030516725.1	Egypt: Alexandria	2020	Blood	blood	-	158	KL49	Ph3
LBMM 6385	GCA_030516795.1	Egypt: Alexandria	2020	Clinical unspecified	swab	-	158	KL49	Ph3
NCGM 306	GCA_016501225.1	Vietnam: Ho Chi Minh	2012	Clinical unspecified		-	215	KL60	Ph3
NCGM 305	GCA_016501245.1	Vietnam: Ho Chi Minh	2012	Clinical unspecified		-	215	KL60	Ph3
DETAB-E227	GCA_017753625.1	China:Hangzhou	2019	Environmental	environment swab	-	309	KL169	Ph4
UV_1268	ERR197569	Vietnam: Ho Chi Minh	2005	Respiratory	VAP	10	575	KL58	Ph4
3_W5.2	GCA_027257355.1	Germany	2019	Environmental	chick-box-paper	-	1554	-[1]	Ph4
AB_347	GCA_003939595.1	Pakistan	2016	Clinical environment	nursing call button in hospital intensive care unit	-	145	KL117	Ph5
AB_349	GCA_003939585.1	Pakistan	2016	Clinical environment	bedside rail in hospital intensive care unit	-	145	KL117	Ph5
XH639	GCA_001863705.1	China: Hangzhou	2014	Respiratory	sputum	-	875	KL117	Ph6
LWSS-5/1-61	GCA_034507315.1	Germany	2019	Environmental	Pig production settings	-	46	KL120	Ph7
MJH_58.1	GCA_026675765.1	Portugal	2005	Respiratory	expectoration	-	46	KL120	Ph7
MJH_195	GCA_026675895.1	Portugal	2009	Respiratory	bronchial secretions	-	46	KL120	Ph7
MJH_58.2	GCA_026675935.1	Portugal	2006	Respiratory	expectoration	-	46	KL120	Ph7
MJH_174	GCA_026675955.1	Portugal	2008	Respiratory	bronchial secretions	-	46	KL120	Ph7
MJH_191	GCA_026675965.1	Portugal	2008	Respiratory	bronchial secretions	-	46	KL120	Ph7
MJH_152	GCA_026676015.1	Portugal	2007	Clinical unspecified	unspecified swab	-	46	KL120	Ph7
316.1	GCA_014893665.1	Portugal: Porto	2015	Clinical unspecified		-	46	KL120	Ph7
MRSN1339	GCA_016538425.2	USA: Washington, DC	2010	Clinical unspecified	Surveillance	-	154	KL58	Ph8
13ARS_MAR0082	GCA_016513555.1	Phillipines		Clinical unspecified		10	2525	KL135	Ph9
U17-HoPe-P3-1	GCA_028438115.1	Germany: Silstedt	2017	Environmental	plant root	-	2416	KL135	Ph10
WU_MDCI_Ab134	GCA_025406495.1	USA: St. Louis	2018	Respiratory	respiratory tract	10	10	KL80	Ph11
SE41055_AB_114	GCA_021571015.1	Serbia: Belgrade	2018	Blood	blood	-	46	KL120	Ph12
SE41035_AB_101	GCA_021571235.1	Serbia: Belgrade	2018	Blood	blood	-	46	KL120	Ph12
SE41005_AB_86	GCA_021571405.1	Serbia: Belgrade	2018	Blood	blood	-	46	KL120	Ph12
PUMA0272	GCA_037724785.1	Singapore	2019	Clinical unspecified		-	216	KL117	Ph13
16-RewurLo-1	GCA_028444085.1	Germany: Loburg	2016	Animal	Lumbricina	-	940	KL120	Ph14
IHIT32682	GCA_026474745.1	Germany	2016	Animal	wound_horse	-	1740	KL120	Ph15
15-7P645-1	GCA_028446045.1	Poland: Opole	2015	Animal	Ciconia ciconia	-	2273	KL58	Ph16
16-118-1	GCA_028444705.1	Poland: Poznan	2016	Animal	Ciconia ciconia	-	2297	KL58	Ph17

[1] No significant match

[2] Incomplete sequence found over multiple contigs

Supplementary Table 3. Details of the prophage carrying *epaA*-*epaB* detected in *A. baumannii* genomes

Prophage number	Sequence length	Family[1]	Complete[2]	Reference isolate	NCBI accession number (base positions)
1	43,065 bp	*Caudoviricetes*	Complete	BAL062	LT594095.1 (2545250-2588314)
2	29,132 bp	*Caudoviricetes*	Incomplete	RES-546	JAMGSJ010000041.1 (1-29132)
3	42,051 bp	*Caudoviricetes*	Complete	NCGM_207	DADAWT010000004.1 (63516-105566)
4	42,792 bp	*Caudoviricetes*	Complete	DETAB-E227	CP072526.1 (1286168-1328959)
5	43,397 bp	*Caudoviricetes*	Incomplete	AB_347	RHYJ01000022.1 (1-43397)
6	40,475 bp	*Caudoviricetes*	Complete	XH639	LYKQ01000058.1 (3718-44192)
7	45,445 bp	*Caudoviricetes*	Complete	LWSS-5/1-61	DASOCS010000001.1 (744757-790201)
8	16,050 bp	*Caudoviricetes*	Complete	MRSN 1339	AAYNSB020000002.1 (334187-350236)
9	41,976 bp	*Caudoviricetes*	Complete	13ARS_MAR0082	DADBIM010000007.1 (6534-48509)
10	17,274 bp	*Caudoviricetes*	Incomplete	U17-HoPe-P3-1	JANIXU010000020.1 (39755-57028)
11	8,153 bp	*Caudoviricetes*	Incomplete	WU_MDCI_Ab134	JAHPSY010000028.1 (1-8153)
12	25,073 bp	*Caudoviricetes*	Incomplete	SE41005_AB_86	JAKHAD010000014.1 (67760-92832)
13	40,392 bp	*Caudoviricetes*	Incomplete	PUMA0272	JBBJSW010000004.1 (1-40392)
14	31,300 bp	*Caudoviricetes*	Incomplete	16-RewurLo-1	JAOWYO010000012.1 (1-31300)
15	38,380 bp	*Caudoviricetes*	Incomplete	IHIT32682	DAKHQI010000028.1 (1-38380)
16	40,029 bp	*Caudoviricetes*	Incomplete	15-7P645-1	JANJDZ010000021.1 (11089-51117)
17	33,062 bp	*Caudoviricetes*	Incomplete	16-118-1	JAOWZG010000007.1 (155500-188561)

[1] Predicted using PhageScope

[2] Determined via alignment with *A. baumannii* A320 (NCBI accession number CP032055.1)

Supplementary Table 4. Primers used in this study.

Primer name	Sequence (5' - 3')	Use
JK0356	CTGATAAATGCTTCAATAATATTGAAAAAGGAAGAGTGTGCAATACGAATGGCGAAAAGC	Amplification of *aacC4* from pSRC119-A/C. Primer includes 23 bp immediately downstream of *aacC4* start, plus 37 bp pUC19 backbone at *bla* promoter
JK0355	CGGAAATGTTGAATACTCATTCAGCCAATCGACTGGCGAG	Amplification of *aacC4* from pSRC119-A/C. Primer includes 20 bp immediately upstream of *aacC4* stop, plus 20 bp pUC19 backbone at *bla* start
JK0385	GGGTGTTGGCGGGTGTCGGGGCTGGCTTAAGGATTTTAACATTTTGCGTTGTTCCAAAAG	Amplification of *ori* from pWH1266. Primer includes 30 bp at *ori* start, plus 30 bp pUC19 backbone immediately downstream of *lacZa*
JK0461	GCGGATAACAATTTCACACAGGAAACAGCTGATCGTAGAAATATCTATGATTATCTTGAA	Amplification of *ori* from pWH1266. Primer includes 30 bp at *ori* end, plus 30 bp pUC19 backbone at *lac* operator
JK0386	GTGGCTTTTTTACGTCTAAAGGAAAACGAAGATCGTAGAAATATCTATGATTATCTTGAA	Amplification of *ori* from pWH1266. Primer includes 30 bp at *ori* end, plus 30 bp sequence downstream of *epaA-epaB* from BAL062
JK0387	TTCAAGATAATCATAGATATTTCTACGATCTTCGTTTTCCTTTAGACGTAAAAAAGCCAC	Amplification of *epaA-epaB* region from BAL062. Primer includes 30 bp sequence downstream of *epaA-epaB* from BAL062, plus 30 bp sequence of pWH1266 *ori* end
JK0388	GCGGATAACAATTTCACACAGGAAACAGCTTGCCGCCTTCGGGCGGTTTTTAACCATTAT	Amplification of *epaA-epaB* region from BAL062. Primer includes 30 bp sequence upstream of *epaA-epaB* from BAL062, plus 30 bp sequence of pUC19 backbone at *lac* operator
JK0389	AGCTGTTTCCTGTGTGAAATTGTTATCCGCTCAC	Amplification of pUC19 backbone to remove MCS and *lacZa*. Primer includes 34 bp of pUC19 sequence at *lac* operator
JK0390	TTAAGCCAGCCCCGACACCCGCCAACA	Amplification of pUC19 backbone to remove MCS and *lacZa*. Primer includes 27 bp of pUC19 sequence immediately downstream of *lacZa*.

Supplementary Table 5. SRA numbers used for phylogenetic analyses

Isolate	Short read accession	Date	Hospital
91_an	ERR197594	2004	Hospital for Tropical Diseases
354_n	ERR263728	2006	Hospital for Tropical Diseases
BAL056	ERR190414	2009	Hospital for Tropical Diseases
BAL064	ERR190417	2009	Hospital for Tropical Diseases
BAL084	ERR190418	2009	Hospital for Tropical Diseases
BAL114	ERR190423	2009	Hospital for Tropical Diseases
BAL346	ERR190491	2011	Hospital for Tropical Diseases
BAL383	ERR190501	2012	Hospital for Tropical Diseases
BAL372	ERR190499	2012	Hospital for Tropical Diseases
BAL369	ERR190498	2012	Hospital for Tropical Diseases
BAL377	ERR190500	2012	Hospital for Tropical Diseases
BAL341	ERR190490	2011	Hospital for Tropical Diseases
BAL350	ERR190493	2011	Hospital for Tropical Diseases
BAL295	ERR190473	2011	Hospital for Tropical Diseases
BAL219	ERR190450	2010	Hospital for Tropical Diseases
BAL230	ERR190454	2010	Hospital for Tropical Diseases
BAL225	ERR190452	2010	Hospital for Tropical Diseases
BAL315	ERR190478	2011	Hospital for Tropical Diseases
BAL238	ERR190457	2010	Hospital for Tropical Diseases
BAL215	ERR190448	2010	Hospital for Tropical Diseases
BAL298	ERR190474	2011	Hospital for Tropical Diseases
BAL128	ERR190425	2010	Hospital for Tropical Diseases
BAL058	ERR190415	2009	Hospital for Tropical Diseases
UV_1897	ERR197570	2007	Hospital for Tropical Diseases
NCGM195	DRR035559	2011	Cho Ray Hospital
NCGM207	DRR035569	2011	Cho Ray Hospital
NCGM194	DRR035558	2011	Cho Ray Hospital
NCGM197	DRR035560	2011	Cho Ray Hospital
NCGM192	DRR035556	2011	Cho Ray Hospital
NCGM211	DRR035573	2011	Cho Ray Hospital

SUPPLEMENTARY FIGURES

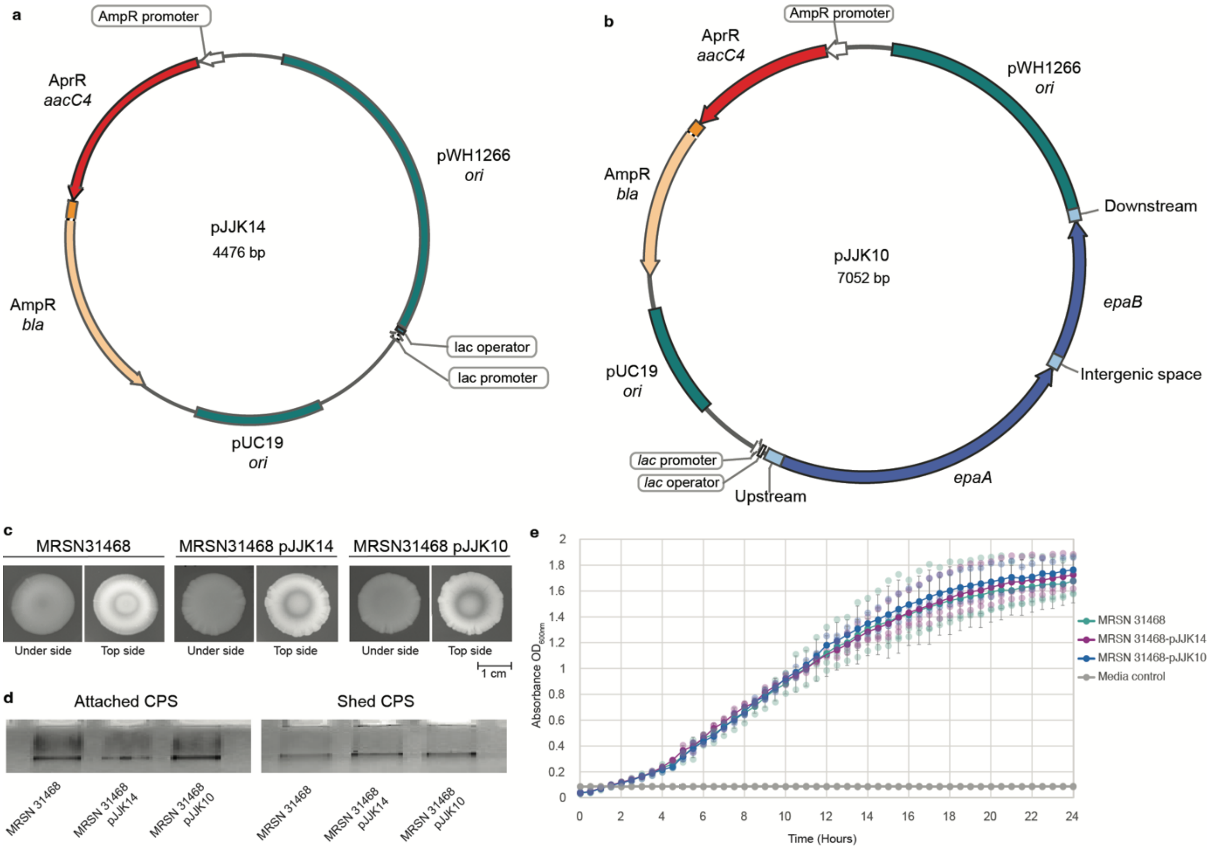

Supplementary Figure 1. Cloning vectors and effect of bacterial growth and CPS production. (**a,b)** Sequence maps of the pJJK14 empty vector (**a**) and pJJK10 vector with *epaA*-*epaB* (**b**) created in Snapgene. Green indicates *ori* regions, red and orange are resistance markers and blue in sequence from BAL062 (NCBI accession number LT594095.1; bases 2549509 to 2552084). **c**, Colony morphology (under side and top side) of MRSN 31468 wildtype, MRSN 31468-pJJK14 and MRSN 31468-pJJK10. Scale bar (1 cm) is shown bottom right of panel. (**d**) CPS produced by MRSN 31468 strains visualised on SDS-PAGE gels stained with Alcian blue. Source data (uncropped gels) is provided in Supplementary Figure 5. (**e**) Growth curves for strains used in this study, measured at OD_{600nm} every 30 minutes for 24 hours. Light coloured points represent measurements for *n*=3 biologically independent samples were used, with the mean represented by dark coloured points showing error bars indicating standard deviation.

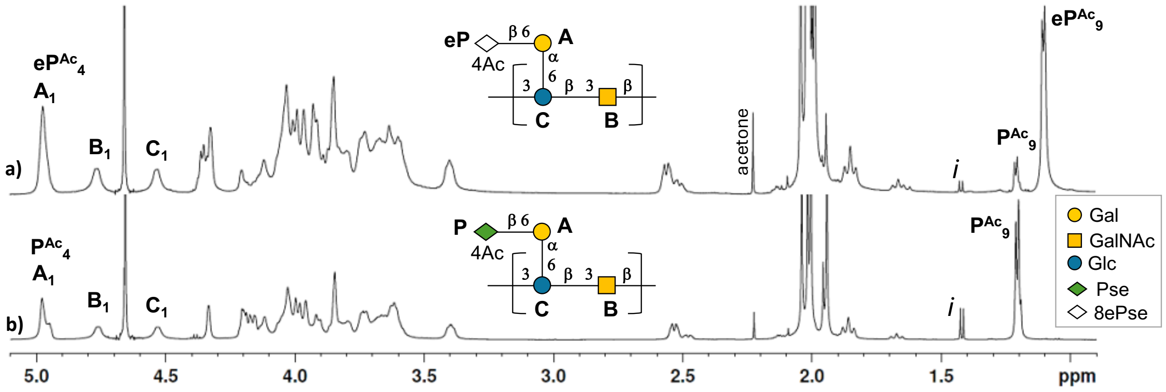

Supplementary Figure 2. ^{1}H-NMR profiles and structures of the capsular polysaccharides from *A. baumannii* strains MRSN 31468 and MRSN 31468-pJJK10 measured at 37°C at 600 MHz. The structure of each CPS is reported above the corresponding proton spectrum, and these structures only differ for the type of nonulosonic acid linked to the α-Gal unit **A**. In both cases these nonulosonic acids are partially acetylated at O-4, as indicated by the upscript "Ac". The two proton spectra have slight differences that are more evident in the high field region at level of the methyl group of the nonulosonic acids, denoted P^{Ac}_{9} for Pse, and eP^{Ac}_{9} for 8ePse. (**a**) proton spectrum of MRSN 31468-pJJK10 CPS that comprises primarily 8ePse (methyl signal at 1.10 ppm) and a minor amount of Pse (methyl signal at 1.20 ppm); (**b**) MRSN31568 CPS proton spectrum. The CPS structures are drawn according to the Symbolic Nomenclature for Glycans, the capital letters nearby each residue reflect those used during the NMR assignment procedure (chemical shifts in Table 2). "*i* " stands for impurity. Source data is available in Supplementary Data 2.

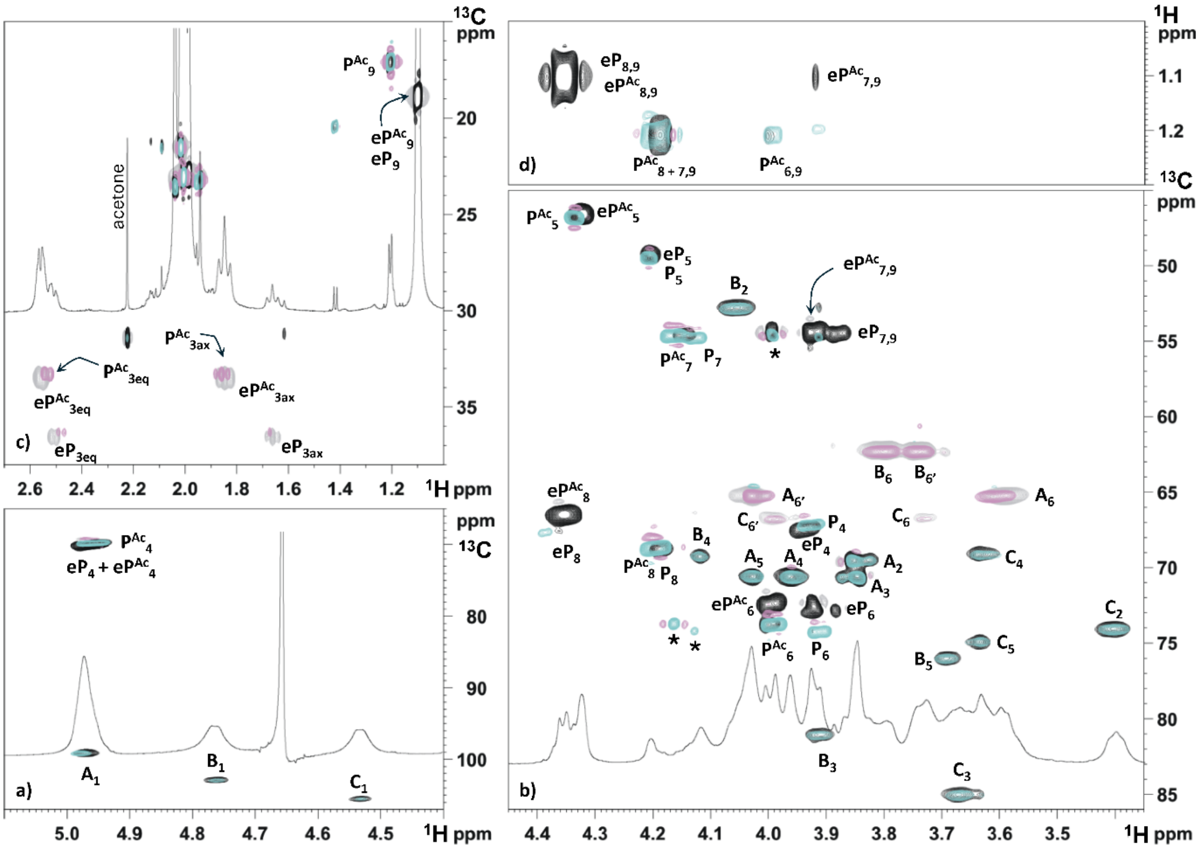

Supplementary Figure 3. 2D NMR spectra recorded for the CPS produced by *A. baumannii* MRSN 31468-pJJK10 and comparison (by overlay) with the spectra of the CPS from MRSN31468 (wildtype). The spectra recorded for MRSN 31468-pJJK10 are traced in black/grey, while those of the strain MRSN31468 are cyan/pink, and the structure of the two CPSs are reported in Supplementary Fig. 2. These CPSs have the same backbone structure and differ for the nature of the NulO. (**a**) Region of the HSQS spectrum detailing the anomeric signals and the C-4 of the NulOs shifted at low field because acetylated; (**b**) HSQC overlay of the carbinolic regions of the two CPSs; (**c**) HSQC overlay of the low field region detailing the 3- and 9-deoxy position of the NulOs of the two CPSs; (**d**) overlay of the TOCSY recorded for two CPSs (MRSN 31468-pJJK10 with black densities; MRSN 31468 in cyan) detailing the correlations between the methyl groups of the NulOs with the protons in the carbinolic region. Densities labeled with "*" are artifacts of the HSQC spectrum known as " COSY effects" that occur between vicinal protons if related by large proton-proton coupling constants. Source data is available in Supplementary Data 2.

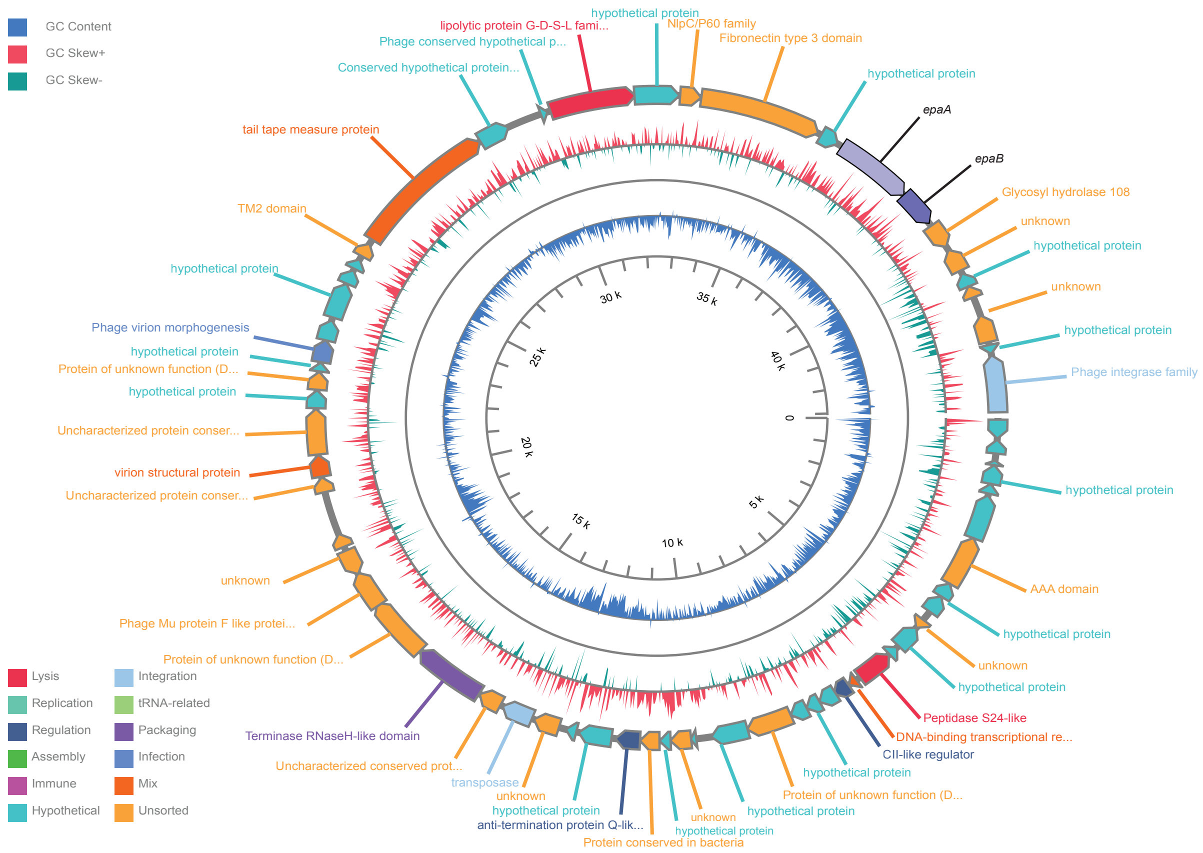

Supplementary Figure 4. Annotation of Prophage 1 genome detected in the BAL062 chromosome. Visualisation constructed using PhageScope, which identifies open reading frames (ORFs; denoted by arrows oriented by transcription direction), assigns predicted functions, and categorizes proteins into functional groups (coloured; scheme bottom left). Base positions, GC content and GC skew are shown by the center rings. The *epaA*-*epaB* genes are indicated (top right).

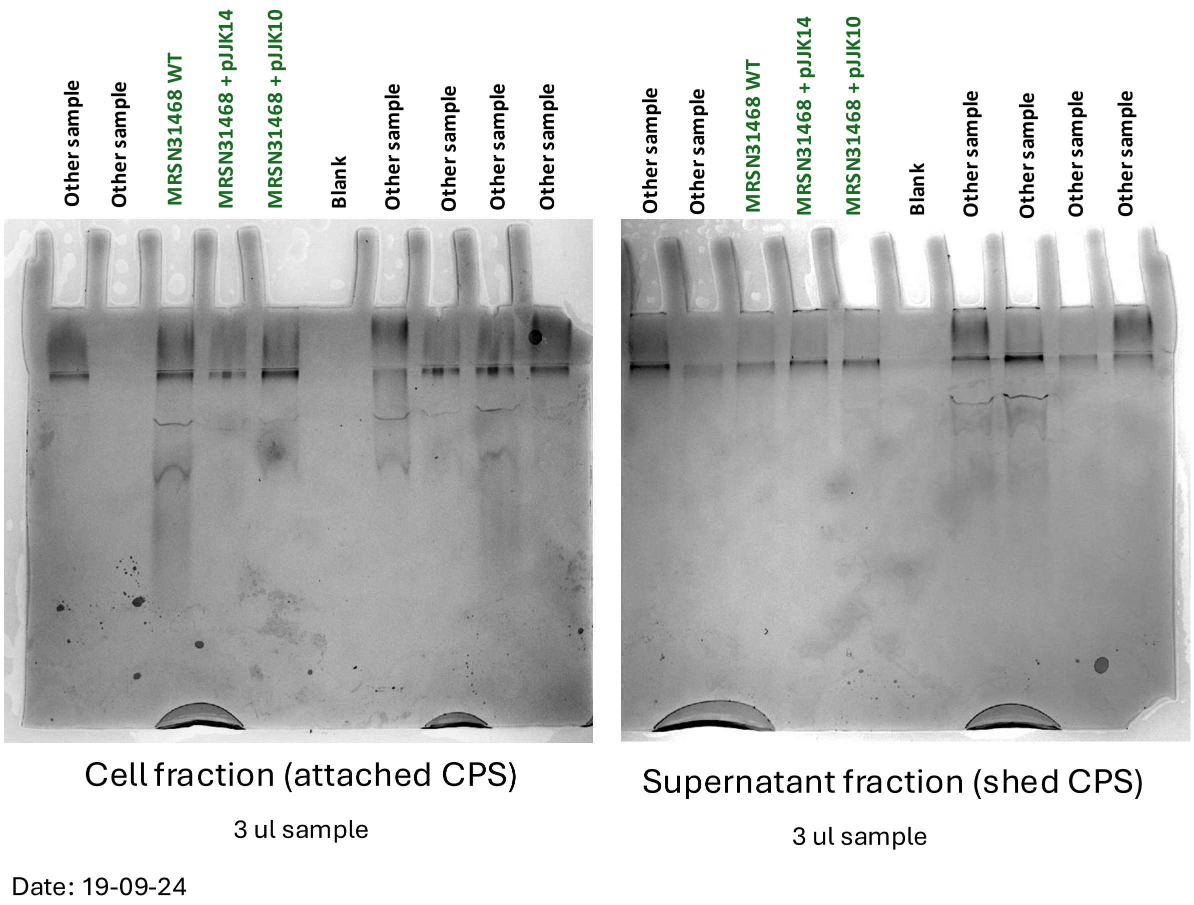

Supplementary Figure 5. Source data for gels shown in Supplementary Figure 1. SDS-PAGE tricine gels (4% stacking and 16% separating) were loaded with 3 ul of sample, stained with Alican blue, and imaged using a ChemiDoc XRS gel imaging system. CPS bands are observed within the stacking gel, predominately at the interface between the stacking and separating gels. Samples relevant to this study are labelled in green font.
