# Supplementary figures and images for: Phage-encoded enzymes found in Acinetobacter baumannii convert pseudaminic acid to 8-epipseudaminic acid
Source: Commun Biol. 2025 May 5;8:700. doi: 10.1038/s42003-025-08114-8 (PMC12053666; doi:10.1038/s42003-025-08114-8)

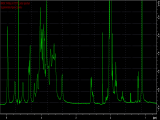

Supplement: Supplementary file 3 — Supplementary Data 2 [file 42003_2025_8114_MOESM3_ESM.zip › FigureS2/1/pdata/1/thumb.png]

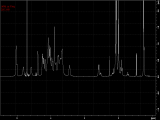

Supplement: Supplementary file 3 — Supplementary Data 2 [file 42003_2025_8114_MOESM3_ESM.zip › FigureS2/2/pdata/1/thumb.png]

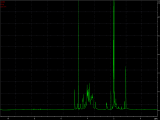

Supplement: Supplementary file 3 — Supplementary Data 2 [file 42003_2025_8114_MOESM3_ESM.zip › FigureS3/1/pdata/1/thumb.png]

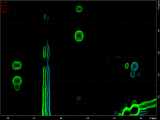

Supplement: Supplementary file 3 — Supplementary Data 2 [file 42003_2025_8114_MOESM3_ESM.zip › FigureS3/4/pdata/1/thumb.png]

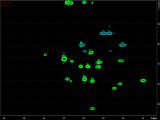

Supplement: Supplementary file 3 — Supplementary Data 2 [file 42003_2025_8114_MOESM3_ESM.zip › FigureS3/3/pdata/1/thumb.png]

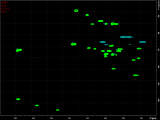

Supplement: Supplementary file 3 — Supplementary Data 2 [file 42003_2025_8114_MOESM3_ESM.zip › FigureS3/2/pdata/1/thumb.png]

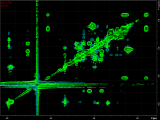

Supplement: Supplementary file 3 — Supplementary Data 2 [file 42003_2025_8114_MOESM3_ESM.zip › FigureS3/5/pdata/1/thumb.png]
