# Supplementary material for: Phage-encoded enzymes found in Acinetobacter baumannii convert pseudaminic acid to 8-epipseudaminic acid
Source: Commun Biol. 2025 May 5;8:700. doi: 10.1038/s42003-025-08114-8 (PMC12053666; doi:10.1038/s42003-025-08114-8)
Supplement: Supplementary file 4 — Description of Additional Supplementary Files [file 42003_2025_8114_MOESM4_ESM.docx]

Description of Additional Supplementary Files

**File name:** Supplementary Data 1

**Description:** Source data for the graph in Fig. S1.

**File name:** Supplementary Data 2

**Description:** Source data for NMR.
